# Redefining shared sanitation

**DOI:** 10.2471/BLT.14.144980

**Published:** 2015-04-28

**Authors:** Thilde Rheinländer, Flemming Konradsen, Bernard Keraita, Patrick Apoya, Margaret Gyapong

**Affiliations:** a Department of Public Health, University of Copenhagen, Øster Farigmagsgade 5, 1014 Copenhagen, Denmark.; b Africa Sanitation Think Tank, 03 BP 7112, Ouagadougou 03, Burkina Faso.; c Dodowa Health Research Center and School of Public Health, University of Ghana, Accra, Ghana.

As the Millennium Development Goals reach their deadline, it is clear that the world is not on track to achieve global sanitation targets. With sanitation trends, global developments and local contexts in mind, it is time to adopt a more flexible approach to achieving universal functional sanitation. By functional sanitation, we mean toilet facilities that protect human health by preventing contamination of the environment with human faecal waste.

According to the latest estimates from the World Health Organization/United Nations Children’s Fund Joint Monitoring Programme for water and sanitation (JMP), 2.5 billion people worldwide do not have access to any type of improved sanitation. To meet the JMP definition of improved sanitation, toilets must be used by only one household, as well as meeting certain design standards that prevent human contact with faeces. Of this 2.5 billion, 732 million use a facility that does not meet minimum hygiene standards and one billion people practise open defecation (i.e. defecation without using a toilet facility).^1^ The remaining group of 784 million people depend on public or other types of shared sanitation facilities as their only sanitation choice. JMP defines shared sanitation as “sanitation of an otherwise acceptable type shared between two or more households.”^1^

The proportion of people depending on shared toilets is higher in the least developed countries (16%) and highest in sub-Saharan Africa, where 19% of the population depends on shared sanitation.^1^ In the same region, a staggering 33% of the urban population depends on shared sanitation, and in 17 sub-Saharan countries the rates of people using shared sanitation is on the increase. In four Asian countries, Bangladesh, China, Mongolia and the Philippines, over 15% of the population depend on shared sanitation – a number that increases daily.^1^

Current definitions do not account for the diversity of shared sanitation: all shared toilet facilities are by default classified as unimproved by JMP because of the tendency for shared toilets to be poorly managed and unhygienic. However, we argue that shared sanitation should not be automatically assumed to be unimproved. We also argue that it is necessary to have a new look at how we define shared sanitation and to use specific subcategories including household shared (sharing between a limited number of households who know each other), public toilets (intended for a transient population, but most often the main sanitation facility for poor neighbourhoods) and institutional toilets (workplaces, markets etc.). This subclassification will identify those depending on household shared sanitation, which we consider to be only a small step away from achieving access to private and improved sanitation. This subcategory of shared sanitation is, therefore, worth discussing in greater detail.

Experiences from Ghana and other sub-Saharan African countries illustrate how household shared sanitation may well fit with culturally acceptable sanitation choices and not necessarily be unhygienic. Indeed, household shared sanitation may be the only realistic option that brings people the important first step up the sanitation ladder from open defecation to a basic level of sanitation.

In Ghana, shared sanitation has in fact contributed immensely to increasing access to sanitation in recent years. From 1999–2012, the usage of shared sanitation facilities increased from 29% to 59%. In the same period, some types of unimproved sanitation designs have been successfully phased out, so that the proportion of people using improved sanitation facilities has also increased. But 19% of the population still practises open defecation and the overall rate of improved sanitation use still stands at a low level of 14%, leaving Ghana far from reaching sanitation development goals.^2^

The success of shared sanitation in Ghana has been attributed to several factors, but notably the planning of living settlements.^3^ The 2008 Ghana Living Standards Survey reports that 79% of Ghanaians live in compound houses rather than self-contained apartments.^4^ Compound houses consist of several households built around a common open area or yard that share utilities like water, electricity and sanitation. In rural areas, compound houses have traditionally hosted multi-generational families, but the structure persists in urban and periurban communities, where it is a practical housing type for migrants and tenants. The majority of compound residents depend on shared sanitation.

Looking more closely at the progress of sanitation along rural–urban divides in Ghana, the importance of shared sanitation is clear. Trends of urbanization and migration are strong in Ghana – as in many other low- and middle-income countries. According to a 2010 census, 50.9% lived in urban areas, with a 7.2% increase in the urban population over just one decade.^5^ From 1990 to 2010, access to shared toilet facilities in these urban areas increased from 46% to 72%.^2^

Two sanitation research projects from southern periurban communities in Ghana indicate that one third of the population would prefer to have shared toilets due to issues of land tenure, financial means and bio-physical factors that limit their ability to invest in and construct single-household toilets.^3^^,^^6^ Land and tenure issues are well known barriers for sanitation in many other sub-Saharan countries, as tenants and settlers are forbidden to build private toilets on land that can be legally and traditionally claimed by others. This is particularly important in urban settings, where huge migrating and poor populations cluster together on limited rented space. Landlords may not see any need to provide tenants with home sanitation facilities and tenants are in no position to demand this – with the result that public sanitation or open defecation are the only options available for them.

In low- and middle-income settings, relatively little is known about local perceptions and cultural barriers for using shared sanitation. Experiences show that crowding, age, gender, privacy, maintenance standards, cleanliness, cost, distance and a range of sociocultural and economic factors can all affect the acceptability of shared toilets.^7^^–^^9^ Recent studies on sharing of sanitation facilities have reported some interesting findings. In poor urban slum communities in Dar es Salaam, the United Republic of Tanzania, shared toilets among families or between landlords and tenants were the most common type of sanitation. In this setting, compared to non-shared facilities, shared facilities were substantially more likely to be functional, safe and robust (in terms of proper facility design, functional condition and waste management systems).^10^ This was attributed to the fact that pooling cash resources to build, maintain and operate a shared facility is easier compared with trying to resolve the issues as a single household. Landlords were also able to obtain higher rents when they offered better quality sanitation facilities to their tenants.^10^ In rural India, members of social sanitation network groups felt a high level of social connectedness which motivated users to manage shared sanitation facilities properly.^11^

Poor tenants in growing cities all over the world are a major user group of shared sanitation with little power to advocate for change. We therefore argue that housing policies and enforcement systems should require landlords to provide proper sanitation.

A recent global review published for the World Bank acknowledges the importance of focusing on shared sanitation – especially in poor urban and periurban communities.^12^ While many challenges remain with communal facilities (operation, maintenance, financial viability, convenience and security) the authors of the review argue that:

“if space allows, but household toilets are not practicable or affordable, shared toilets reserved for the use of small, self-selected groups *may* be preferable to communal facilities, and the sense of ownership created *may* encourage users to keep the facilities clean.”^12^(Emphasis added).

As the Millennium Development Goals reach their deadline, it is clear that the world is not on track to achieve the global sanitation targets. Given the sanitation trends, global developments and local contexts in mind, it is time to adopt a more flexible approach to achieving universal functional sanitation by adopting a more nuanced definition of shared sanitation.

Key sanitation stakeholders and donors should recognize the potential of household shared sanitation as an important driver behind sanitation progress in African and Asian high-density areas and low-income populations. Accepting household shared sanitation as a suitable toilet type could have major implications. This would legitimize innovative funding mechanisms, shared maintenance schemes and upgrading of large numbers of existing shared toilets to acceptable standards.

We argue that the focus for future sanitation programmes should be on improving the hygienic standards of shared facilities to a level that satisfies and protects sanitation users – irrespective of the toilet design. If well managed, household shared sanitation can be a feasible, economical, practical and socially acceptable choice for millions of sanitation users.
